# Possible immunotherapeutic potentiation with D-Fraction in prostate cancer cells

**DOI:** 10.1186/1756-8722-1-25

**Published:** 2008-12-04

**Authors:** Paul Pyo, Brandon Louie, Srinivas Rajamahanty, Muhammad Choudhury, Sensuke Konno

**Affiliations:** 1 Department of Urology, New York Medical College, Valhalla, NY 10595, USA

## Abstract

**Background:**

Prostate cancer remains the most common malignancy among elderly men and the second leading cause of cancer death in the United States. Although several conventional therapies are currently available, they have a low efficacy and the more effective treatment modalities need to be established. Interferons (IFNs) are one of such options known as immunotherapy and demonstrated their antitumor effects on certain cancer types. Yet such antitumor activity should be improved or potentiated to have the satisfactory outcomes. In fact, *combination *therapy has been proposed as an alternative approach and is being underway in human and animal studies. Accordingly, we studied whether the combination of IFN-α and D-fraction (PDF), a bioactive mushroom extract, might potentiate anticancer activity of IFN-α in prostate cancer PC-3 cells *in vitro*.

**Results:**

Potential effects of recombinant IFN-α_2b _(0–100,000 IU/ml), PDF (0–1,000 μg/ml), or their combinations were assessed on the growth of PC-3 cells at 72 h. Cell cycle analysis using a flow cytometer and Western blot analysis were performed to explore antiproliferative mechanism of these agents. The dose-dependent study showed that IFN-α_2b _up to 20,000 (20 K) IU/ml had no significant effects, but >60% growth reduction was attained ≤50 K IU/ml. Similarly, PDF showed no effects up to 250 μg/ml but ~65% growth reduction was seen at 1,000 μg/ml. When IFN-α_2b _and PDF were combined, a relatively low concentration (10 K IU/ml) of IFN-α_2b _and PDF (250 μg/ml) resulted in a ~65% growth reduction. This was accompanied by a G_1 _cell cycle arrest, indicated by cell cycle analysis. Western blots also revealed that the G_1_-specific cell cycle regulators, CDK2, CDK4, CDK6, cyclin D_1_, and cyclin E, had been significantly (>60%) down-regulated in IFN/PDF-treated cells.

**Conclusion:**

The combination of IFN-α_2b _(10 K IU/ml) and PDF (250 μg/ml) is capable of inducing a ~65% reduction in PC-3 cell growth. This appears to be due to a synergistic potentiation of two agents, leading to a G_1 _cell cycle arrest. Thus, it is conceivable that PDF may potentiate IFN-α_2b _activity, improving immunotherapy for prostate cancer.

## Background

Current therapy for prostate cancer (CaP), the most common malignancy in elderly men in the United States [1], is directed at exploitation of the androgen-dependent state of prostatic cancer cells. Various antiandrogens and leuteinizing hormone-releasing hormone (LHRH) agonists are useful for blocking the availability of androgen to the cancer cells [2]. However, the efficacy of these drugs is of limited duration, and patients experience an almost inevitable progression of their cancers to the fatal androgen-independent state [3]. To develop an alternative approach for controlling or preventing such disease progression, it demands in searching for agents/drugs that could effectively regulate the CaP proliferation.

Interferons (IFNs) are known to trigger multiple cellular responses including antiviral activity, growth inhibition, cell differentiation and immunoregulation [4]. Many studies have also focused on the potential antitumor effects of IFNs using both *in vitro *and *in vivo *cancer models [5]. For instance, IFNs have been widely used as immunotherapy for urological malignancies including prostate, bladder, and renal carcinomas [6-8]. Compared to chemotherapy, less or moderate side effects of IFNs have also been shown to be beneficial to cancer patients. Some encouraging data from such IFN monotherapy have been reported [9], although they are yet somewhat inconsistent and conflicting. In addition, particularly in clinical CaP cases, IFN therapy has several drawbacks such as high cost and repeated administration [6]. These disadvantages thus limit its use in clinical practice, and further exploration of improved treatment modality, e.g. combination therapy, is required.

The D-fraction (PDF), the unique proteoglucan extracted from maitake mushroom (*Grifola frondosa*), is the acid-insoluble, alkali-soluble and hot water-extractable fraction [10]. It structurally consists of either β-1,6-linked glucan with β-1,3 branches or β-1,3 glucan branched with β-1,6 glucosides, having a molecular weight of ~1 × 10^6 ^dalton [10]. PDF has been commercially available for a variety of medical and scientific research, and a number of published and unpublished studies have thus far suggested the immunomodulatory and antitumor activities of PDF [11-13]. It has been shown in an animal model that PDF was capable of activating immune-competent cells such as natural killer cells and cytotoxic T-cells with a concomitant increase in interleukin-1 production [11], indicating stimulation of immune responses. A preventive or inhibitory activity of PDF on carcinogenesis and metastasis has also been demonstrated in mice [12], suggesting its antitumor activity. Moreover, a chemosensitizing effect of PDF has been postulated on conventional anticancer drugs being currently used [13].

Accordingly, we are interested in investigating whether the combination of IFN-α_2b _and PDF may have the potentiated growth inhibitory effects on prostatic cancer cells *in vitro*. Such studies may provide us with useful information on the improved efficacy of IFN therapy on prostate cancer.

## Results

### Effects of IFN-α_2b _or PDF on PC-3 cell growth

**Figure 1:**
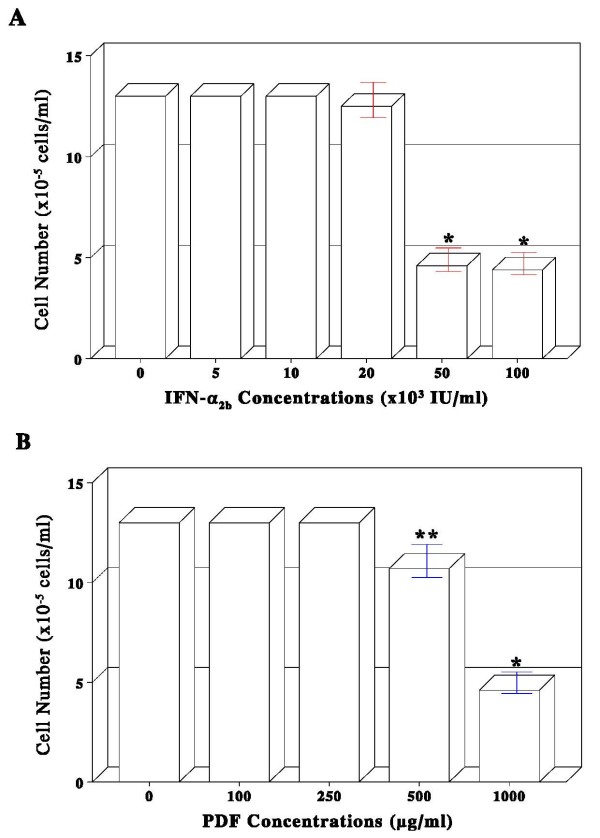
**Dose-dependent effects of IFN-α _2b _or PDF on PC-3 cell growth**. PC-3 cells were cultured with varying concentrations of either IFN-α_2b _(0–100,000 IU/ml) or PDF (0–1,000 μg/ml) as indicated, and viable cell numbers in IFN-α_2b_-treated (**A**) or PDF-treated (**B**) cases were determined at 72 h. All data represent mean ± SD (standard deviation) from three separate experiments (*p < 0.02; **p < 0.08).

To examine the possible effects of individual IFN-α_2b _or PDF on PC-3 cell proliferation, cells were cultured with varying concentrations of IFN-α_2b _(0–100,000 = 100 K IU/ml) or PDF (0–1,000 μg/ml) for 72 h. Such a dose-dependent study showed that IFN-α_2b _had no apparent effects up to 20 K IU/ml but induced >60% growth reduction at 50 K and 100 K IU/ml (Fig. 1A). Similarly, no effects of PDF was seen up to 250 μg/ml but a marginal (10–20%) and significant (~65%) growth reduction was observed at 500 and 1,000 μg/ml, respectively (Fig. 1B). Thus, these studies demonstrate that both IFN-α_2b _and PDF are capable of inhibiting PC-3 cell growth, although PDF required rather a high concentration (1,000 μg/ml) to be effective.

### Synergistic growth inhibitory effects of IFN-α_2b _and PDF

**Figure 2:**
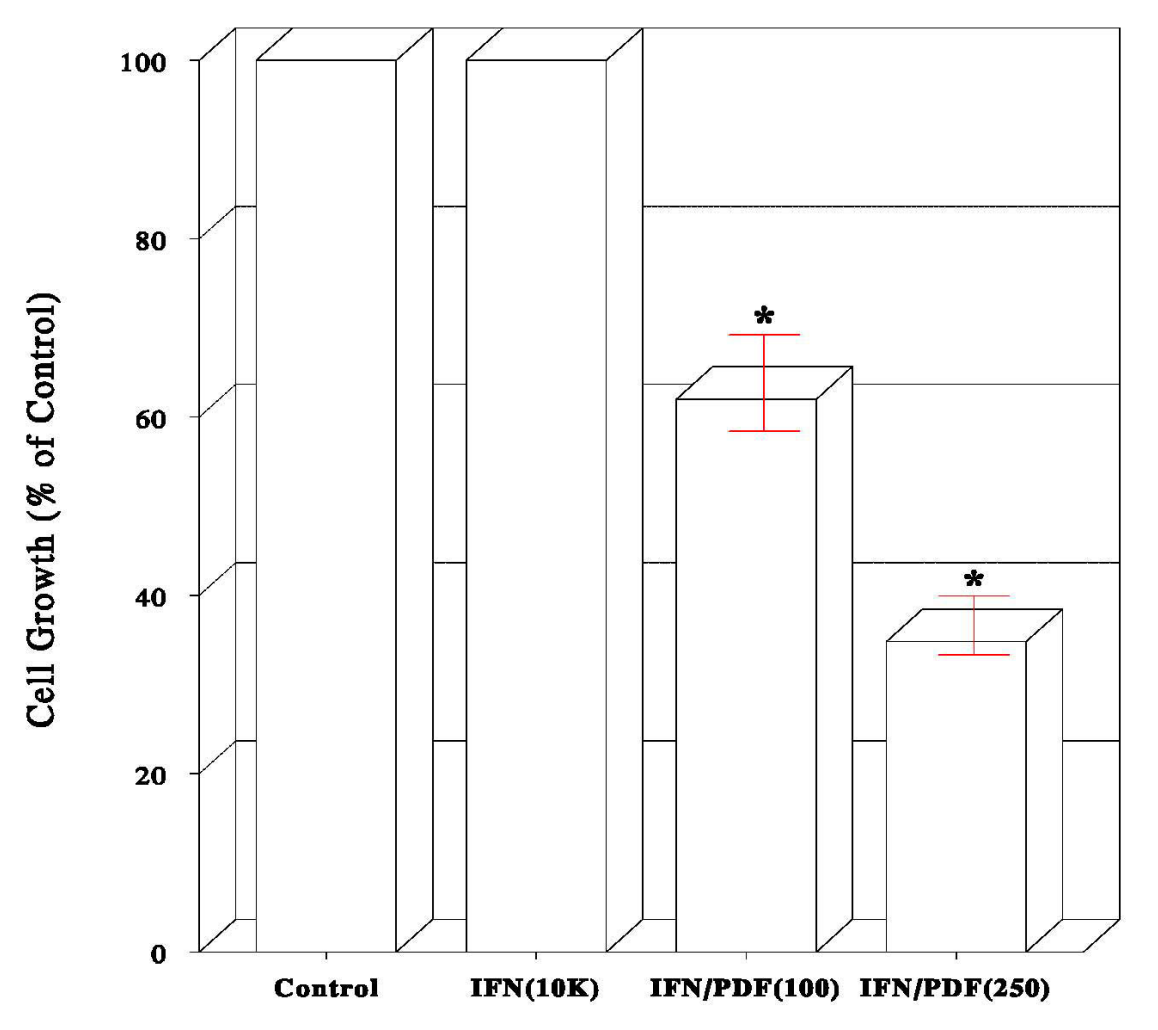
**Effects of combinations of IFN-α _2b _and PDF on cell growth**. Cells were cultured with varying concentrations of IFN-α_2b_/PDF combination for 72 h, and cell growth was assessed by the % of viable cell number relative to that in control (100%). Cell growth in control, IFN-α_2b _(10 K IU/ml)-treated, or IFN-α_2b _(10 K)/PDF (100 μg/ml)-treated, or IFN-α_2b _(10 K)/PDF (250)-treated cells is shown. The data are mean ± SD from three independent experiments (*p < 0.05).

To examine whether combinations of IFN-α_2b _and PDF may exhibit the enhanced growth inhibitory effects, the varying concentrations of IFN-α_2b _and PDF were combined and their effects on PC-3 cell growth were assessed. Such results showed that combinations of 10 K IU/ml of IFN-α_2b _and 100 or 250 μg/ml of PDF resulted in nearly 40% or 65% growth reduction, respectively (Fig. 2). These enhanced inhibitory effects are most likely attributed to a synergistic potentiation of two agents, because the given concentrations of IFN-α_2b _(10 K IU/ml) and PDF (100 or 250 μg/ml) by itself had no such effects (Fig. 1AB). Thus, IFN-α_2b _and PDF appear to work synergistically.

### Effects of IFN-α_2b_, PDF, or their combination on cell cycle

**Figure 3:**
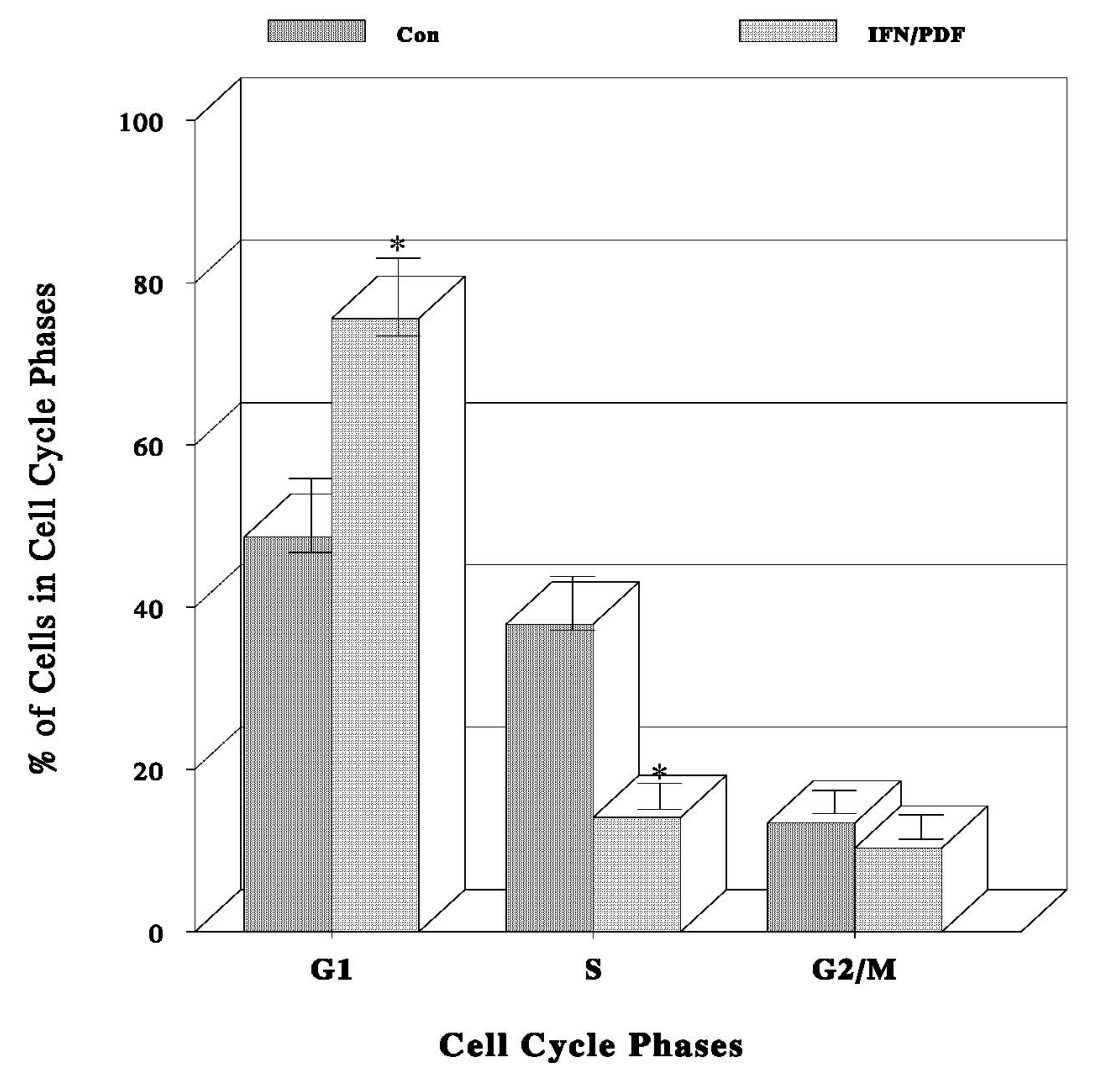
**Cell cycle analysis**. Cells were cultured with IFN-α_2b _(10 K IU/ml), PDF (250 μg/ml), or their combination for 72 h, and they were subjected to cell cycle analysis as described in Materials and Methods. IFN-α_2b _or PDF alone had little effects on cell cycle phase distribution similar to that in control cells. However, the IFN/PDF combination resulted in significant changes (*p < 0.05) in cell populations in the G_1 _and S phases compared to controls, as shown here.

To better understand the underlying mechanism of such a synergistic growth inhibition induced by the IFN-α_2b_/PDF combination, their possible effects on the cell cycle were explored next. Cells were treated with IFN-α_2b _(10 K IU/ml), PDF (250 μg/ml), or their combination for 72 h, and they were subjected to cell cycle analysis using a flow cytometer. IFN-α_2b _or PDF alone had little effects similar to cell cycle phase distribution in control cells; however, the IFN-α_2b_/PDF combination caused an ~63% decrease in cell number in the S phase with a concomitant 55% increase in the G_1_-phase cell population compared to those in controls (Fig. 3). These results indicate that the IFN-α_2b_/PDF combination causes a blockage of cells entering from the G_1 _to the S phase, increasing cell number in the G_1 _phase. This accumulation of cells in the G_1 _phase is known as a G_1 _cell cycle arrest [14], which feasibly leads to the growth inhibition.

### Down-regulation of cell cycle regulators by IFN-α_2b_/PDF combination

**Figure 4:**
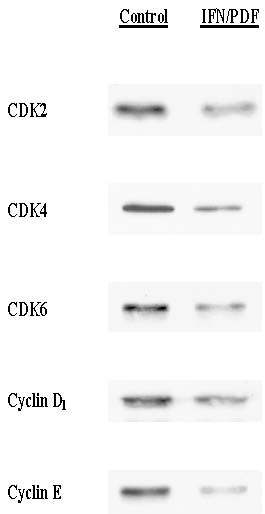
**Western blot analysis**. After cells were treated with or without the combination of IFN-α_2b _(10 K IU/ml) and PDF (250 μg/ml) for 72 h, cell lysates (7 μg) obtained from control and the IFN-α_2b_/PDF-treated cells were analyzed for CDK2, CDK4, CDK6, cyclin D_1_, and cyclin E using Western blots. Significantly (>60%) reduced expressions of all these regulators following the IFN/PDF treatment are apparent on the blots.

To confirm a G_1 _cell cycle arrest induced by the IFN-α_2b _(10 K IU/ml)/PDF (250 μg/ml) combination, we also examined its effects on the specific cell cycle regulators for the G_1_-S phase transition such as CDK2, CDK4, CDK6, cyclin D_1_, and cyclin E [14]. After 72-h treatment, cell lysates were prepared and subjected to Western blot analysis, as described in Materials and Methods. Cellular expressions of these cell cycle regulators following IFN-α_2b_/PDF treatment were all significantly reduced by >60% (quantitated by densitometric scanning), compared to those in controls (Fig. 4). Such a down-regulation of these cell cycle "promoters" (to be more properly described) further supports a blockage of G_1_-S phase transition. Taken together, these studies are highly suggestive that a G_1 _cell cycle arrest is the critical event taking place in the IFN-α_2b_/PDF-induced growth inhibition.

## Discussion

IFNs belong to the family of cytokines and are capable of activating a cascade of intracellular pathways that regulate cell growth/differentiation and also produce antiviral and immunological responses [4,15]. Particularly, the antitumor potential of IFNs gained a great attention and has been extensively investigated for over two decades. Some early studies showed that IFNs had induced regression of tumors in a significant number of patients with metastatic breast cancer, low-grade lymphoma, and multiple myeloma [16]. However, the efficacy of IFNs on tumor regression was also found to vary with cancer types [17], and some data from specific IFN *monotherapy *indeed showed such discrepancy. Moreover, a high cost and repetitive administration of IFN (monotherapy) somewhat limit its clinical utility. Accordingly, reducing a cost while improving the efficacy of IFNs, "combination" therapy has been proposed and promoted.

In the present study, we explored such combination therapy as an alternative approach for prostate cancer immunotherapy; i.e. combination of IFN-α_2b _and D-fraction (PDF). Dose-dependent studies showed that IFN-α_2b _≥ 50 K IU/ml or PDF at 1,000 μg/ml was capable of inducing >60% growth reduction in prostate cancer PC-3 cells (Fig. 1). Moreover, a ~65% growth reduction was attained with the combination of 10 K IU/ml IFN-α_2b _and 250 μg/ml PDF (Fig. 2). This augmented growth inhibition results conceivably from a synergistic potentiation of two agents, because neither 10 K IU/ml IFN-α_2b _nor 250 μg/ml PDF *alone *has any growth inhibitory activity (Fig. 1). Thus, the relatively low concentrations of IFN-α_2b _and PDF when combined are required for their potentiated antiproliferative effects. In other words, to attain the same growth inhibitory effect (~65%) induced by 50 K IU/ml of IFN-α_2b _alone, merely "1/5^th^" (10 K IU/ml) of that IFN-α_2b _would be needed when combined with PDF. It is then plausible that PDF may not only help potentiate IFN-α_2b _activity but also help cut the cost down.

We next examined the effects of IFN-α_2b_/PDF combination on the cell cycle regulation in order to explore the growth inhibitory mechanism. Cell cycle analysis revealed a ~63% decrease in the S-phase cell number with a concomitant 55% increase in the G_1_-phase cell number following the treatment of IFN-α_2b_/PDF combination (Fig. 3). The resulting "G_1 _cell accumulation" is termed a G_1 _cell cycle arrest, accounting in part for the ultimate growth cessation. In addition, the expressions of specific G_1 _cell cycle regulators, such as CDK2, CDK4, CDK6, and cyclins D_1_/E, were all markedly (>60%) down-regulated (Fig. 4). Thus, these findings suggest that the growth inhibitory action of IFN-α_2b_/PDF combination may target primarily the G_1_-S phase transition in the cell cycle, resulting in a G_1 _arrest.

Yet, it should be also noted that IFNs are known to modulate many proteins and enzymes [18], particularly specific *protein kinases *acting on the signal transduction pathway for cell proliferation and/or differentiation. In other words, IFNs can regulate cell growth through the signal transduction mediated by these protein kinases. Additionally, it has been documented that IFNs could induce DNA fragmentation, leading to an accumulation of small or low-molecular-weight DNA [19]. This may imply activation of a specific protein kinase called double-stranded DNA-dependent protein kinase (DNA-PK), which requires small double-stranded DNA for its activation [20]. DNA-PK is also believed to play an important role in the cell cycle regulation [21]. These information further suggest that our IFN-α_2b_/PDF combination may affect certain protein kinase(s), triggering the specific cascade events (via the signal transduction) on the cell cycle to ultimately cease cancer cell growth. Therefore, such biochemical studies are undoubtedly required and being underway in our laboratory.

In addition, it is important to further investigate whether the enhanced growth inhibitory effect of IFN-α_2b_/PDF combination observed in this *in vitro *study might be also demonstrated in animal study (*in vivo*). Such study would then allow us to assess the actual efficacy of IFN-α_2b_/PDF combination on prostate tumor grown in mice and to determine the effective or tolerable physiological concentrations of these agents. This will be conducted shortly as our Phase II study.

Furthermore, the safety of IFN-α_2b _or PDF in human use would be certainly concerned. IFN-α_2b _has been often used in immunotherapy for various cancer patients and its concentrations up to 5 × 10^6 ^IU have been shown to be relatively safe and tolerable in those with prostate cancer [9,22]. It eventually needs to be determined how the effective concentration of 10 K IU/ml IFN-α_2b _(in combination with PDF) in this study would be extrapolated to actual patients. For PDF, early animal and clinical studies ascertained the safety of PDF without any side/adverse effects [13]. This was further supported by the fact that the U.S. Food and Drug Administration (FDA) had exempted D-fraction from a Phase I toxicology study. The FDA has also approved PDF for an Investigational New Drug (IND) application to conduct a Phase II pilot study on patients with advanced breast and prostate cancer [23]. Although such clinical trials are currently in progress, the effective concentrations of PDF yet remain to be established. Taken together, our next animal study is crucial and indisputably required for confirming the safety of IFN-α_2b _and PDF and also obtaining valid information on their effective and tolerable physiological concentrations. It may then help lead us to an ultimate clinical trial in the future.

## Conclusion

In summary, the combination of IFN-α_2b _and PDF demonstrates a synergistic antiproliferative activity on prostate cancer PC-3 cells. This potentiated growth inhibition results primarily from a G_1 _cell cycle arrest. Therefore, the low-dose IFN-α_2b_/PDF combination may provide an alternative, improved immunotherapy for prostate cancer, implying its clinical utility/application. It is promising but further studies are yet required.

## Methods

### Cell culture

The human prostate cancer PC-3 cells, derived from a patient with bone metastasis, were obtained from the American Type Culture Collection (Rockville, MD). Cells were maintained in RPMI-1640 medium containing 10% fetal bovine serum, penicillin (100 U/ml), and streptomycin (100 μg/ml). Routinely, culture medium was changed every 3 to 4 days and the passage of cells was performed weekly. For experiments, cells were seeded in T-75 flasks or 6-well culture plates at the initial cell density of 1 × 10^5 ^cells/ml and were cultured with recombinant interferon-α_2b _(IFN-α_2b_; Schering Corp., Kenilworth, NJ), D-fraction (PDF; Maitake Products, Inc., Paramus, NJ) or their combinations. Cell numbers were then assessed at specified times using the trypan blue exclusion method.

### Cell cycle analysis

A FACScan flow cytometer (Becton-Dickinson, San Jose, CA), equipped with a double discrimination module, was employed for cell cycle analysis. Approximately 1 × 10^6 ^cells were resuspended in 500 μl of propidium iodide solution (20 μg/ml propidium iodide, 0.2 mg/ml RNase, 0.2 mg/ml EDTA, 0.5% NP-40) and incubated at room temperature for 1 h. Ten thousand nuclei were analyzed for each sample, and CellFit software was used to quantify cell cycle compartments and estimate cell cycle phase fractions.

### Western blot analysis

Cell pellets from control and IFN-α_2b_/PDF-treated cells were resuspended in cell lysis buffer and cell lysates were prepared by freeze-thaw three times in liquid nitrogen. The Western blot procedure essentially followed the protocol described previously [24]. Briefly, an equal amount of proteins (7 μg) from control and IFN-α_2b_/PDF-treated cell lysates was resolved by 10% SDS-PAGE (SDS-polyacrylamide gel electrophoresis) and transferred to a nitrocellulose membrane. The blot was first incubated for 90 min with the primary antibodies against CDK2, CDK4, CDK6, cyclin D_1_, or cyclin E (Santa Cruz Biotechnology, Santa Cruz, CA), followed by incubation with the appropriate secondary antibody conjugates for 30 min. The specific immunoreactive proteins were then detected by chemiluminescence, following a vender's protocol (Kirkegaard and Perry Laboratories, Gaithersburg, MD), and quantified using a scan densitometer (Silk Scientific, Oregon, UT).

### Statistical analysis

All data are presented as the mean ± SD (standard deviation), and statistical differences between groups were assessed with the unpaired Student's *t *test. A value of *p *< 0.05 is considered to be significant.

## Competing interests

The authors declare that they have no competing interests.

## Authors' contributions

PP is a primary investigator in charge of performing all experiments and drafting the manuscript; BL and SR serve as assistants for PP to help set up and run experiments (cell culture, flow cytometer, and Western blots); MC is the department chairman, providing us with all his support for this project; and SK is responsible for designing experiments, analyzing the data (and statistical analysis), and editing/finalizing the manuscript. All authors read and approved the final manuscript.
